# HIResist: a database of HIV-1 resistance to broadly neutralizing antibodies

**DOI:** 10.1093/bioinformatics/btae103

**Published:** 2024-02-29

**Authors:** Milind Misra, Jeffy Jeffy, Charis Liao, Stephanie Pickthorn, Kshitij Wagh, Alon Herschhorn

**Affiliations:** Division of Infectious Diseases and International Medicine, Department of Medicine, University of Minnesota, Minneapolis, MN 55455, United States; Division of Infectious Diseases and International Medicine, Department of Medicine, University of Minnesota, Minneapolis, MN 55455, United States; Division of Infectious Diseases and International Medicine, Department of Medicine, University of Minnesota, Minneapolis, MN 55455, United States; Division of Infectious Diseases and International Medicine, Department of Medicine, University of Minnesota, Minneapolis, MN 55455, United States; Theoretical Division, Los Alamos National Laboratory, Los Alamos, NM 87545, United States; Division of Infectious Diseases and International Medicine, Department of Medicine, University of Minnesota, Minneapolis, MN 55455, United States; Institute for Molecular Virology, University of Minnesota, Minneapolis, MN 55455, United States; Institute for Engineering in Medicine, University of Minnesota, Minneapolis, MN 55455, United States; Center for Genome Engineering, University of Minnesota, Minneapolis, MN 55455, United States; Microbiology, Immunology, and Cancer Biology Graduate Program, University of Minnesota, Minneapolis, MN 55455, United States; The College of Veterinary Medicine Graduate Program, University of Minnesota, Minneapolis, MN 55455, United States; Molecular Pharmacology and Therapeutics Graduate Program, University of Minnesota, Minneapolis, MN 55455, United States

## Abstract

**Motivation:**

Changing the course of the human immunodeficiency virus type I (HIV-1) pandemic is a high public health priority with approximately 39 million people currently living with HIV-1 (PLWH) and about 1.5 million new infections annually worldwide. Broadly neutralizing antibodies (bnAbs) typically target highly conserved sites on the HIV-1 envelope glycoproteins (Envs), which mediate viral entry, and block the infection of diverse HIV-1 strains. But different mechanisms of HIV-1 resistance to bnAbs prevent robust application of bnAbs for therapeutic and preventive interventions.

**Results:**

Here we report the development of a new database that provides data and computational tools to aid the discovery of resistant features and may assist in analysis of HIV-1 resistance to bnAbs. Bioinformatic tools allow identification of specific patterns in Env sequences of resistant strains and development of strategies to elucidate the mechanisms of HIV-1 escape; comparison of resistant and sensitive HIV-1 strains for each bnAb; identification of resistance and sensitivity signatures associated with specific bnAbs or groups of bnAbs; and visualization of antibody pairs on cross-sensitivity plots. The database has been designed with a particular focus on user-friendly and interactive interface. Our database is a valuable resource for the scientific community and provides opportunities to investigate patterns of HIV-1 resistance and to develop new approaches aimed to overcome HIV-1 resistance to bnAbs.

**Availability and implementation:**

HIResist is freely available at https://hiresist.ahc.umn.edu/

## 1 Introduction

Approximately 39 million people live with HIV-1 (PLWH) worldwide as of the end of 2022 (https://www.who.int/). Without treatment, human immunodeficiency virus type I (HIV-1) infection leads to gradual decrease of CD4+ T cells and to acquired immunodeficiency syndrome (AIDS) in most patients. Current antiretroviral therapy is highly efficient and decreases HIV-1 viral load to undetectable levels in most treated PLWH, but therapy requires life-long adherence, due to a latent HIV-1 reservoir (Chomont *et al.* 2009, Bertagnolli *et al.* 2020, Jiang *et al.* 2020, Ratnapriya *et al.* 2021, Bellini *et al.* 2022, Lopez *et al.* 2022) and potentially low-level viral replication (Wietgrefe *et al.* 2022), that is associated with long-term adverse effects (Friis-Møller *et al.* 2010). Thus, an effective HIV-1 vaccine (Kwong and Mascola 2018, Stephenson *et al.* 2020, Ratnapriya *et al.* 2022) and cure strategies (Herschhorn *et al.* 2010, Ho *et al.* 2013, Wang *et al.* 2018) are both still needed to halt the progress of the HIV-1 pandemic.

HIV-1 envelope glycoproteins (Envs) mediate viral entry and are the sole target of neutralizing antibodies (Robey *et al.* 1985, Kwong *et al.* 1998, Ahmed *et al.* 2023). Interactions of HIV-1 Envs with the CD4 receptor on target cells trigger conformational transitions to an open Env state that is associated with structural rearrangements and relatively short-lived activation state (Harris *et al.* 2020). CD4-bound (open) state exposes the coreceptor binding site and facilitates Env binding to the CCR5 or CXCR4 coreceptor. Subsequent interactions of gp41 with the cellular membrane lead to the fusion of the viral and cellular membranes and mediate the entry of HIV-1 into target cells (Alkhatib *et al.* 1996, Dragic *et al.* 1996, Feng *et al.* 1996, Trkola *et al.* 1996, Furuta *et al.* 1998, Koshiba and Chan 2003). Either spontaneously or in response to CD4 binding, HIV-1 Envs can transition from a closed (State 1) to an open (State 3) conformation through an obligatory intermediate (State 2) (Herschhorn *et al.* 2016, 2017). The frequency of Env transitions between conformational states likely depends on the architecture and metastability of the Envs of each specific HIV-1 strain with typically infrequent transitions detected for Envs of primary HIV-1 strains (e.g. HIV-1_JRFL_) compared to Envs of lab adapted strains (Munro *et al.* 2014). Major determinants of HIV-1 Env function and conformational state depend on the specific amino acid sequence of each HIV-1 Envs. Moreover, changes of specific amino acids can be detrimental for Env function (Alsahafi *et al.* 2018) while other changes can shift the distributions of HIV-1 Env conformations (Herschhorn *et al.* 2016, Herschhorn and Sodroski 2017, Ratnapriya *et al.* 2020, Kirschman *et al.* 2022, Vilmen *et al.* 2022, Parthasarathy *et al.* 2023).

Broadly neutralizing antibodies (bnAbs) target vulnerable sites on HIV-1 Envs that are critical for virus entry and typically highly conserved in different strains [some target residues are less conserved; for example the V3-glycan, bnAb targeting, N332 is conserved among only ∼75% of M-group HIV-1 strains and mostly absent from HIV-1 strains that belong to clade AE (Stephenson *et al.* 2020, Jeffy *et al.* 2023)] (Walker *et al.* 2009, 2011, Wu *et al.* 2010, Zhou *et al.* 2010, Huang *et al.* 2012, 2016, Haynes *et al.* 2019). As a result, specific bnAbs efficiently neutralize diverse HIV-1 strains and provide opportunities to develop new therapeutic and preventive strategies (Haynes *et al.* 2019). Most bnAbs that target the CD4-binding sites (CD4bs) and those targeting the V1/V2 loop of gp120 prefer to neutralize the closed Env conformation of primary strains, while most bnAbs that target the gp41 membrane external proximal region (MPER) neutralize more efficiently Envs that are more open (Herschhorn *et al.* 2014, 2016, 2017, Flemming *et al.* 2018). In addition, some bnAbs can target equally well different Env conformations and efficiently block viral entry. Several ongoing and completed clinical trials have studied the effects of bnAbs on HIV-1 prevention and on the efficiency of bnAb immunotherapy (Bar *et al.* 2016, Bar-On *et al.* 2018, Corey *et al.* 2021, Juelg *et al.* 2022). These studies provide important information and guidance for HIV-1 vaccine design and development as well as for understanding the potential side effects, mode of administration, and the mechanisms of bnAb action during immunotherapy treatment. Notably, these studies highlight potential direct and indirect mechanisms of HIV-1 resistance to bnAbs, some of which have been already documented in multiple *in vitro* experiments (Herschhorn *et al.* 2011, Yen *et al.* 2014, Lynch *et al.* 2015, Bar *et al.* 2016, Cale *et al.* 2020, Cervera *et al.* 2021, Herschhorn 2023, Mazurov and Herschhorn 2023). Thus, combining Env sequence analysis and experimental approaches to understand the different pathways of HIV-1 resistance to bnAbs is beneficial for future robust application of bnAbs for medical interventions. In addition, this approach could provide insights into the biology of HIV-1 Env function and conformation.

The Los Alamos National Laboratory (LANL) HIV Sequence Database (www.hiv.lanl.gov) provides a variety of valuable data and computational tools to analyse HIV-1 nucleotide and amino acid sequences. In particular, CATNAP (Compile, Analyze and Tally NAb Panels) (Yoon *et al.* 2015) allows to analyse Env sequences of different HIV-1 strains in the context of nAb panels. While instrumental and widely used to analyse HIV-1 sensitivity and resistance to antibodies, a more focused database that provides improved and user-friendly tools to align and annotate Env sequences, to cluster Env sequences according to their resistance profile, to compare antibody cross-sensitivity, and to highlight Envs of special interest is still missing. Here, we have developed a specialized database, HIResist (HIV-1 Resistance to bnAbs), to analyse patterns of HIV-1 resistance to different bnAbs. HIResist is freely available online (at https://hiresist.umn.edu) and is a comprehensive online resource with analysis and visualization tools designed to support the HIV-1 research community and the public. In the subsequent sections, we describe the design and implantation, features, and conclusions of our efforts related to the HIResist database.

## 2 Design and implementation

HIResist is a Flask web application with Gunicorn production server and Apache reverse proxy and is written in Python (50%) and HTML/CSS/JavaScript (50%). Version control is managed by private GitHub repositories. The HIResist web server resides on a Linux virtual machine having eight 2.20 GHz Xeon E5-260 processors and 16 GB RAM. Data retrieved from the CATNAP database (https://www.hiv.lanl.gov/content/index) (Yoon *et al.* 2015) are stored in a periodically updated SQLite database while signature information is stored in a text file.

HIResist was developed in several sequential steps to allow continuous improvements of design and/or visualization. The first step was a simple terminal-based Python program that allowed automatic retrieval and local storage of CATNAP data. For a given, user-specified bnAb, this program enabled display of stored HIV-1 strains in separate resistant and sensitive classes based on a user-specified IC_50_/IC_80_ threshold. The second step was the development of a graphical user interface for the terminal-based program. This version of the program had a menu bar, tool bar, an information box, and a central scrollable viewing area. Not only did these early standalone versions of HIResist guide the visual arrangement of the database’s design elements, but they also served as a laboratory for developing the algorithms that currently drive the various tools on the website.

## 3 Features

HIResist retrieves sequence and neutralization data from public databases such as GenBank (https://www.ncbi.nlm.nih.gov/genbank/), and the Los Alamos National Laboratory HIV databases database (https://www.hiv.lanl.gov/content/index) (Yoon *et al.* 2015). In addition, we routinely add new Env sequences from current and completed clinical trials. HIResist is a searchable database and includes several bioinformatic tools: bnAb reactivity, Env strain sensitivity, bnAb cross-sensitivity plots, and bnAb signatures, which are amino acids/glycans of HIV-1 Envs that are statistically associated with either resistance or sensitivity of HIV-1 to bnAbs (Gnanakaran *et al.* 2010, Bricault *et al.* 2019).

### 3.1 bnAb reactivity

**Figure 1.**
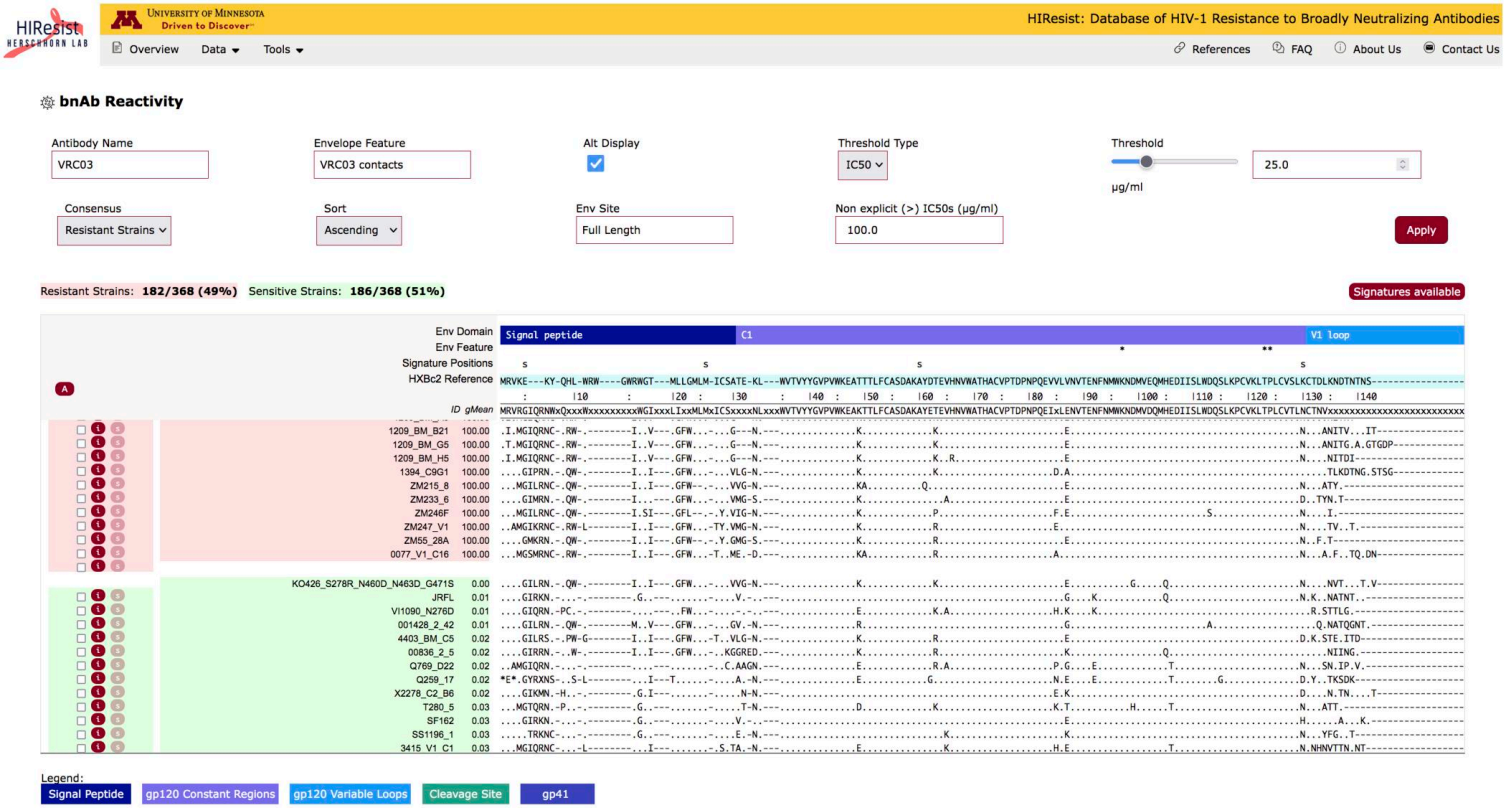
bnAb reactivity compares resistant (top) and sensitive (bottom) Envs to user-specified antibodies at selected threshold.

bnAb Reactivity interface analyzes the database for resistant and sensitive HIV-1 Envs to a specified antibody at a user-selected threshold (e.g. IC_50_ > 50 µg/ml). The antibody is selected in an auto-complete field labeled “Antibody Name” (Fig. 1) and the user can select either IC_50_ or IC_80_ as the threshold for resistance and specify the threshold value on a continuous scale (in µg/ml). The tool then displays the alignment of Env sequences of the members of each group (resistant Envs with red background and sensitive Envs with green background).

**Figure 2.**
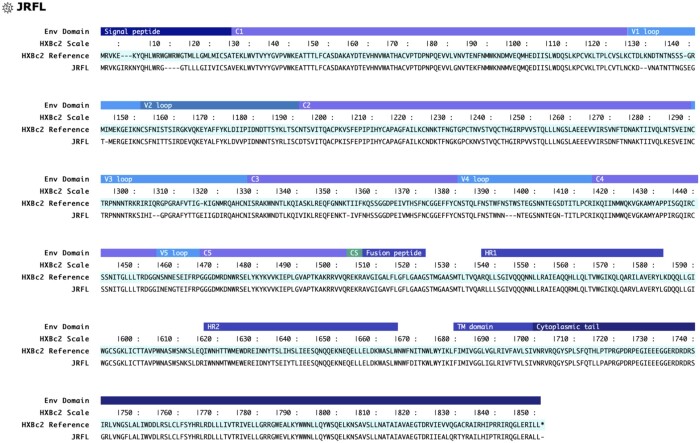
Detailed view of specific Env sequence and features is available from every alignment by double clicking on the specific Env sequence.

The antibody-specific multiple sequence alignment (MSA) displayed is adjusted from the MSA for all HIV-1 Envs in CATNAP (Yoon *et al.* 2015). Since most queries will display only a subset of Envs sequences, which were tested for the specific antibody, the antibody-specific MSA is a gap-adjusted subset of the CATNAP MSA (i.e. unnecessary gaps, which were originally incorporated due to the alignments of all sequences, are removed). Double clicking on a specific sequence opens a new browser tab containing details about the selected Envs (Fig. 2). The HXBc2 sequence is used as a reference for both the MSA and numbering convention of the amino acid positions in the MSA.

When data from multiple assays are available for specific Envs, the geometric mean (gMean) values are also provided in the display. Otherwise, the gMean reflects the results of a single report. The gMean of results from multiple assays is calculated by the following equation:

$$\sqrt[{n}]{X_{1}*X_{2}*\ldots *X_{n},}$$

where X = IC_50_ or IC_80_; and n = number of assays.

We found a significant challenge, in some cases, for calculating/reporting accurate gMean values. Several experiments have reported non-explicit IC_50_/IC_80_ values as “> X µg/ml”, where X = maximum concentration tested in the viral neutralization assay. Classification of these specific Envs as resistant/sensitive or calculating gMean are possible only with some assumptions (e.g. changing the “>” value to be equal to the maximum concentrations tested), some of which may potentially introduce some bias to the analysis.

For example, if IC_50_ of strain A > 10 µg/ml and IC_50_ of strain B = 12.5 µg/ml for a specific bnAb than classification of two strains is possible only after adjusting the IC_50_ of strain A to IC_50_ = 10 µg/ml or any other used-defined value. However, in this case a user-selected threshold of 11 µg/ml will determine that stain A is sensitive (in case IC_50_ was set to 10 µg/ml) and strain B is resistant to the specific bnAb, which is probably not accurate. As a result of these complications, gMean calculation of HIResist currently sets all greater than (“>”) values to a default value of 100 µg/ml and changes all less than (“<”) values to equal (“=”) values. Nevertheless, HIResist provides users with the flexibility to modify the default value for greater than (“>”) values and a similar option for values that were reported as less than (“<”) specific concentration will be implemented in the future; these changes are expected to be determined by the users according to their analysis assumptions and aims.

The “Alt Display” checkbox displays a convenient view to visualize sequence comparisons, with a default set to the alternative display in which identical amino acids are displayed as dashes (“-”) below the parent sequence. The MSA in this view displays amino acids that are different from the HXBc2 reference sequence with the single-letter amino acid convention and all amino acids identical to those of HXBc2 reference are marked as dots. The top panel of the display includes annotations of major Env domains and a bar for displaying user-selected Env features (such as contacts, escape mutations, residue predictions, signature predictions, etc.) denoted as “*” that are entered in the auto-complete field “Envelope Feature”. Users can customize their alignment view by displaying the sequences in ascending/descending order based on IC_50_/IC_80_ values and by generating consensus using resistant and/or sensitive strains. Additionally, users can view sequences by selecting specific Env domains or entering their custom range of HXBc2 reference number using the “Custom” option in the auto-complete field “Env Site” (Fig. 1). When Env signatures are available for the selected antibody: (i) the “Signatures available” button is active and lists all the sensitive and resistant signature positions identified for the antibody and (ii) the top panel shows the signature positions (marked with “s”).

**Figure 3.**
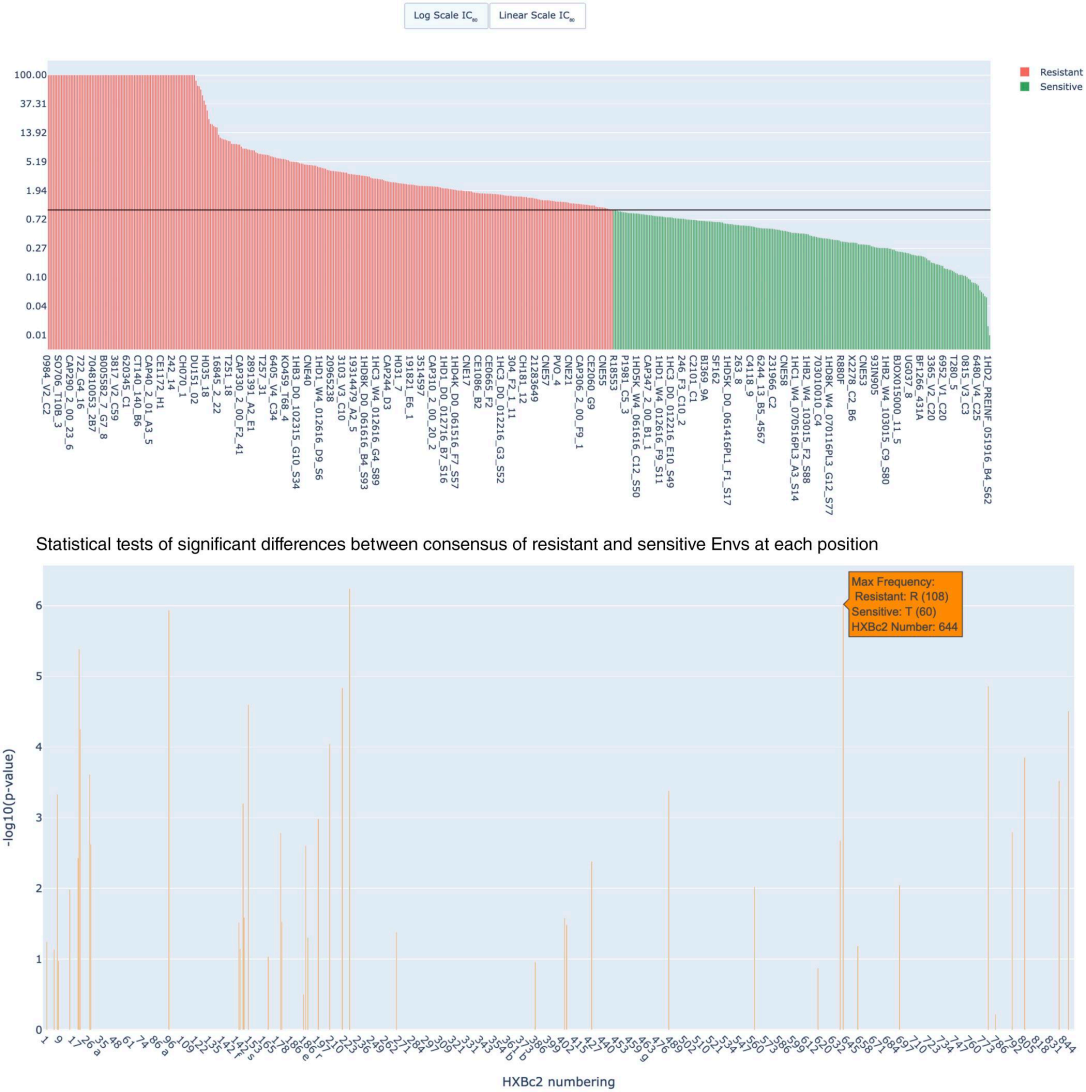
bnAb reactivity generates graphical distribution of Env susceptibility (top) and statistical tests for significance of each consensus amino acid at each position (bottom) of resistant and sensitive Envs. *P*-value bars (bottom panel) show estimate of the appropriate statistical test (Chi-squared test and Fisher’s exact test). Hovering on a specific bar shows the consensus amino acids among resistant and sensitive Envs and the exact position according to HXBc2 numbering.

Two additional plots are displayed below the MSA (Fig. 3): (i) a bar chart of sorted gMean values displayed either at a logarithmic (default) or linear scale and (ii) a bar chart that provides a quantitative statistical analysis of the differences between the consensus of the resistant and sensitive strains at each position. The algorithm assesses each position of the Env sequence and identifies the consensus amino acids in each of the two groups (resistant and sensitive Envs). If the consensus amino acid of the two groups is different, HIResist calculates the prevalence of consensus resistant amino acid among (i) the sensitive Envs and (ii) resistant Envs and similarly the prevalence of consensus sensitive amino acid in the two groups at this position. HIResist then estimates statistically significant differences between the prevalence of a consensus (resistant) amino acid versus consensus (sensitive) amino acid in the groups at each position. We used Fisher’s exact test when all criteria for this test are met and Chi-squared test otherwise. The bar plot displays the negative logarithm of the *P*-value of the related statistical test. The plot is interactive and hovering over each bar displays the predominant amino acid in both the resistant and sensitive groups at that position, along with the corresponding HXBc2 numbering. These plots are generated using plotly and provide options to view the plots in different scales (linear or logarithmic), zoom (in and out), select data, and download the plots as .png file format.

### 3.2 Strain sensitivity

**Figure 4.**
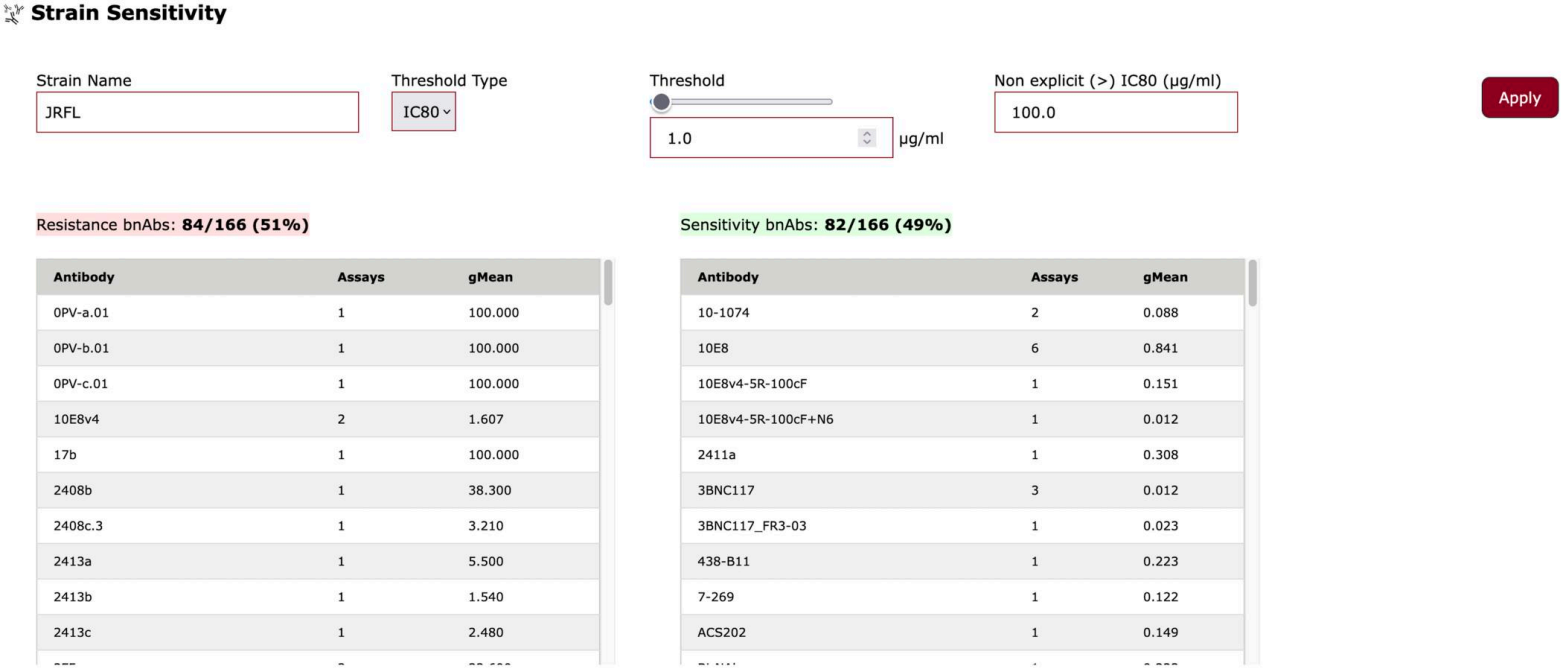
Strain sensitivity identifies and displays neutralizing and non-neutralizing bnAbs/Env ligands (e.g. CD4-Ig) for a user-selected HIV-1 Envs.

Strain sensitivity generates a profile of neutralizing and non-neutralizing ligands/bnAb based on user-specified HIV-1 strain, which is entered in the auto-complete field “Strain Name”, and on a user-selected threshold (Fig. 4). Similar to the bnAb reactivity, the user can select either IC_50_ or IC_80_ as the threshold type and enter a threshold value (in µg/ml) to use for the classification. For consistency, current analysis displays all IC_50_/IC_80_ values with greater than (“>”) values as 100 µg/ml by default and changes all less than (“<”) values to equal (“=”) values, with a user-defined option to set greater than (“>”) values to a specific value. A similar option for values that were reported as less than (“<”) specific concentration planned to be implemented in the future; user-selected values should be based on the specific analysis assumptions and aims.

### 3.3 Cross-sensitivity

**Figure 5.**
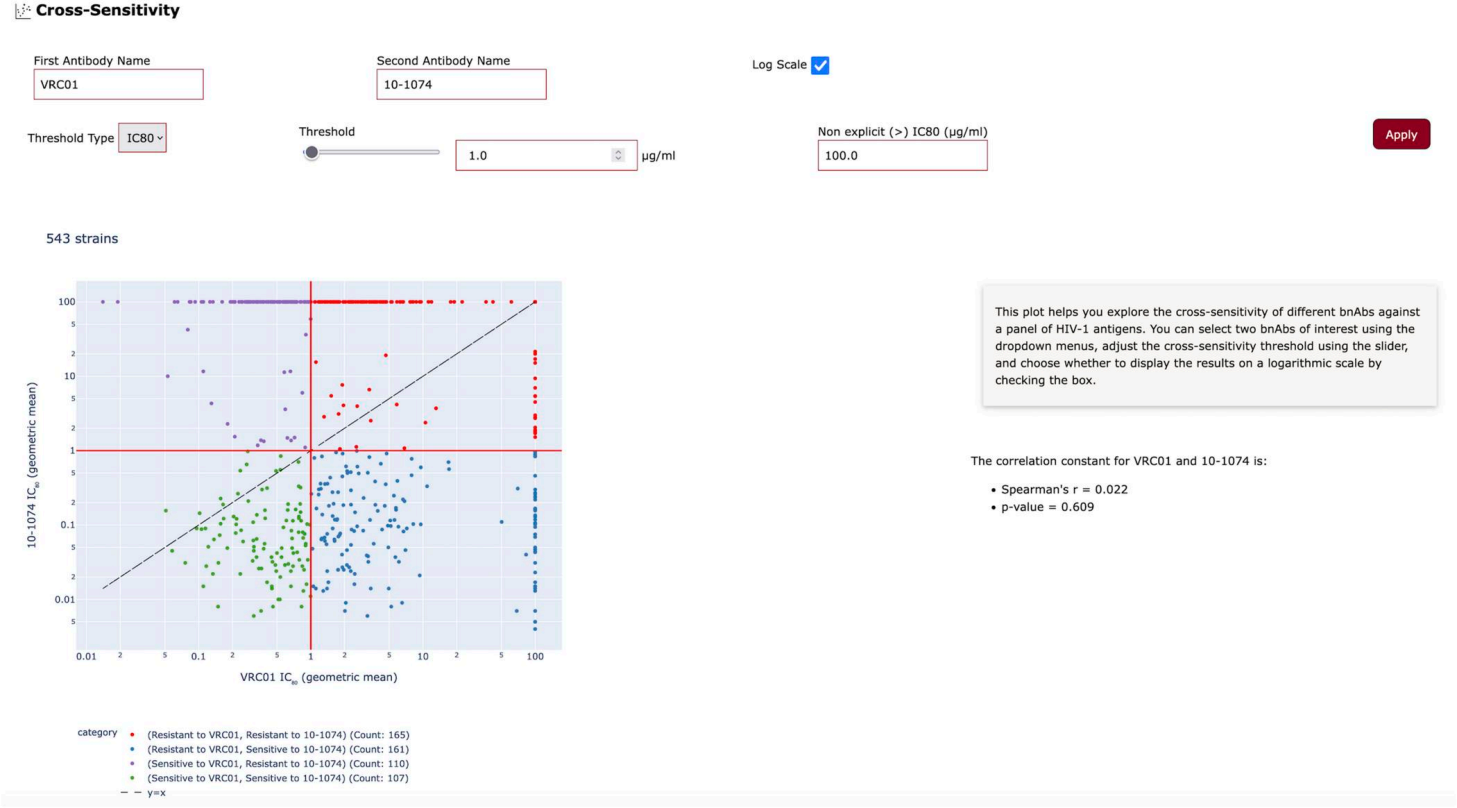
Cross-sensitivity generates plots of the sensitivity of all Envs to a pair of user-selected antibodies/ligands. Four regions of sensitivity/resistance profiles can be defined on the plot.

Cross-sensitivity generates a plot allowing users to explore the cross-sensitivity of HIV-1 Envs in the database to a pair of bnAbs/Env ligands (e.g. CD4-Ig) (Fig. 5). Users select two bnAbs/Env-ligands of interest using the auto-complete fields, a threshold type and value (in µg/ml), and choose whether to display the results on a logarithmic or linear scale. As explained above, IC_50_s of some viral neutralization assays were reported with non-explicit values (e.g. “>”) and they are currently set to 100 µg/ml (for greater than values) unless otherwise specified by the user or to the lowest value reported (for less than values). However, we plan to add to these plots the option to display these values using different marker(s) to allow users to distinguish between explicit and non-explicit inhibition values. Four quadrants on the cross-sensitivity plots define the resistance/sensitivity profile of HIV-1 Envs in each quadrant and are color-coded accordingly: upper-right quadrant—HIV-1 Envs resistant to both bnAbs/Env ligands, lower-left quadrant—HIV-1 Envs sensitive to both bnAbs/Env ligands, and upper-left/lower-right quadrants—HIV-1 Envs that are resistant to one bnAb/Env ligand and sensitive to the other bnAb/Env ligand. Hovering the mouse on a specific point (Envs) displays the specific Envs and gMean values for both bnAbs/Env ligands. The cross-sensitivity tool provides spearman’s rho, *P*-value, and a linear line (y = x; dashed line) to better analyse the relationship between the two antibodies.

### 3.4 Search database

**Figure 6.**
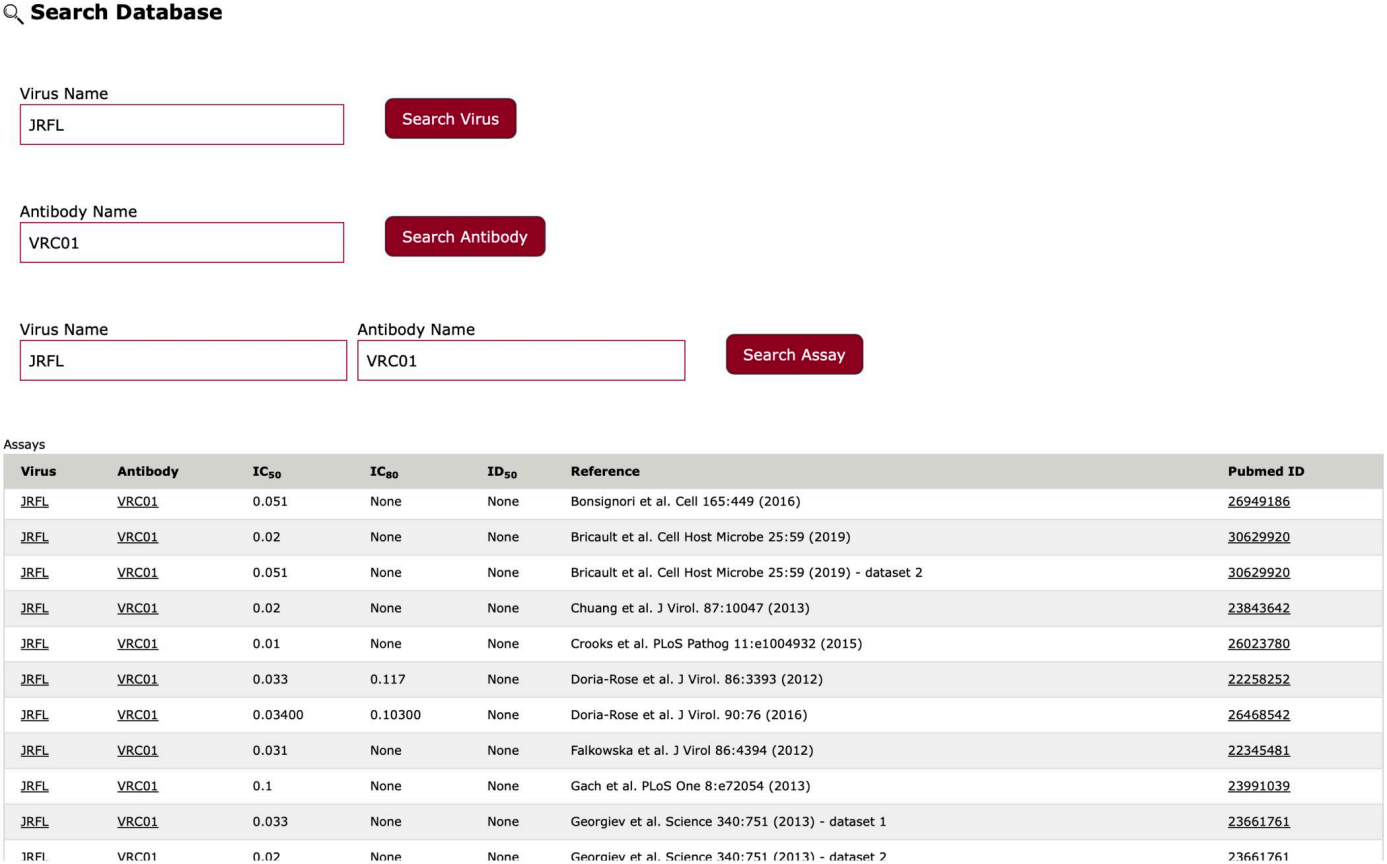
Search database lists all assays in the database that are related to specific HIV-1 strain and/or specific antibody/Env ligands.

The HIResist database contains a search engine to retrieve data at three levels using auto-complete fields (Fig. 6). A search for an HIV-1 strain by the name of the strain directs the user to a web page with strain details including alignment with HXBc2 reference sequence, subtype, country/year of isolation, GenBank accession, alternative used names, nucleotide and amino acid sequences, and assay information. Similarly, a search for antibody directs the user to a web page with details of the antibody including binding type, structure if available, PubMed ID of the article in which antibody isolation was described, alternative used names, and assay information. An example of results of a search for a specific HIV-1 strain (HIV-1_JRFL_) and a specific bnAb (VRC01) is shown in Fig. 6 that presents experimental inhibition data calculated in different experiments and PubMed IDs for related publications.

### 3.5 HIV-1 Env resistant to multiple bnAbs/resistant HIV-1 Envs of interest

**Figure 7.**
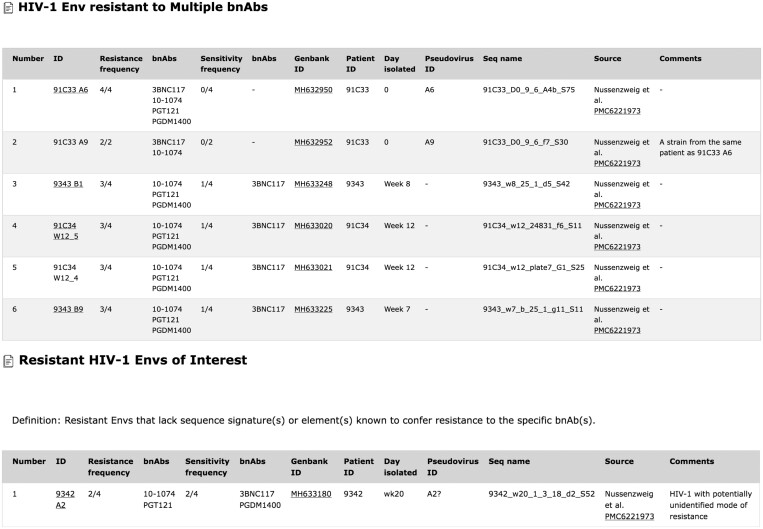
HIV-1 Env resistant to multiple bnAbs and resistant HIV-1 Envs of interest.

In addition to bioinformatic tools listed above, HIResist provides lists of HIV-1 Env resistant to multiple bnAbs and HIV-1 Envs of interest (Fig. 7). We listed in the first group HIV-1 strains (or Envs) that are resistant to multiple bnAbs and can still efficiently enter target cells or were developed in and isolated from patients undergoing bnAb immunotherapy. We anticipate that future indications that any of these strains can easily spread in humans will classify them as of high public health concern. The list of resistant HIV-1 Envs of interest contains variants (or Envs) that exhibit an unusual mechanism of resistance that cannot be explained by a simple change in the antibody epitope. These two lists are routinely updated to include new strains as they are identified and become available from different sources, including completed and ongoing clinical trials.

## 4 Conclusion

HIResist is a new database with a collection of bioinformatic tools that has been developed to assist the analysis and visualization of HIV-1 Env data in the context of resistance and sensitivity to bnAbs. In addition to the tools outlined above for studying bnAb reactivity, strain sensitivity, and bnAb cross-sensitivity, several graphical interfaces are available including heatmaps generated for clustering selected sets of HIV-1 strains and antibodies, pairwise and multiple sequence alignment tools with output displayed in a standard HIResist format, and visualization of resistance/sensitivity signatures of HIV-1 Envs. Furthermore, HIResist can be linked to or work together with other LANL bioinformatic tools that analyse HIV-1 Env sequences and bnAb neutralization data. Potential workflows could involve downloading and using HIResist analyses as inputs for LANL tools. For example, a user could combine analysis of a particular set of Envs by HIResist with a prediction of best 2 bnAb combination to target these sequences by LANL CombiNAber (www.hiv.lanl.gov/content/sequence/COMBINABER/combinaber.html) (Wagh *et al.* 2016). In another example, LANL GenSig (https://www.hiv.lanl.gov/content/sequence/GENETICSIGNATURES/gs.html) (Bricault *et al.* 2019) could be used to identify Env signatures associated with IC_80_ above a threshold for a given bNAb that was identified by HIResist analysis.

We continue to develop new tools to allow robust data analysis of resistance/sensitivity signatures of HIV-1 strains and to visualize patterns of resistance by alignments, heatmaps and resistance trees. We also plan to add several new interfaces including tools for comparison of bnAb resistance/sensitivity in HIV-1 strains from different populations of PLWH (e.g. drug users or elite controllers) and tools for assessment of emerging HIV-1 strains. HIResist is being developed to encourage engagement and exploration of these highly relevant data by the broader scientific community without the need for programming expertise.
